# MRI-based morphometric analysis of the patellofemoral joint: diagnostic modeling of knee pathologies in adolescents

**DOI:** 10.3389/frai.2026.1808422

**Published:** 2026-05-22

**Authors:** Dusan Spasic, Goran Djuricic, Jelena Djokic Kovac, Bojan Bukva, Vladimir Radlovic, Milos Maletic, Stanislav Rajkovic, Marko Radulović

**Affiliations:** 1 Faculty of Medicine, University of Belgrade, Belgrade, Serbia; 2 Department of Pharmacology, Clinical Pharmacology, and Toxicology, Faculty of Medicine, University of Belgrade, Belgrade, Serbia; 3 Department of Diagnostic Imaging, University Children's Hospital, Belgrade, Serbia; 4 Clinic for Digestive Surgery, University Clinical Centre of Serbia, Belgrade, Serbia; 5 Orthopedic Surgery and Traumatology Department, University Children's Hospital, Belgrade, Serbia; 6 Serbian Institute of Sports and Sports Medicine, Belgrade, Serbia; 7 Institute for Orthopaedics “Banjica”, Medical Faculty, University of Belgrade, Belgrade, Serbia; 8 Department of Experimental Oncology, Institute for Oncology & Radiology of Serbia, Belgrade, Serbia

**Keywords:** adolescent knee pathology, diagnostic performance, machine learning ensemble models, MRI-based morphometry, patellar retinacular lesions, patellofemoral joint

## Abstract

**Introduction:**

To evaluate whether routinely measured MRI-based patellofemoral joint morphometric parameters can support diagnostic modeling of selected adolescent knee pathologies and to compare a conventional multivariable logistic regression baseline with machine-learning approaches.

**Methods:**

This retrospective single-center pilot diganostic modeling study included 168 adolescents (97 girls, 71 boys, mean age 15.5 ± 1.7 years) who underwent knee MRI between January 2018 and December 2024 because of anterior knee pain or suspected patellofemoral structural abnormality. Thirteen patellofemoral morphometric parameters were measured by two radiologists. Three binary MRI endpoints were modeled: composite chondromalacia, a composite endpoint of ACL injury or patellar bone bruise, and patellar retinacular lesion. Baseline multivariable logistic regression was compared with machine-learning approaches using chronological training, validation, and test splits. Additional gradient-boosting comparators were evaluated, and uncertainty was quantified using bootstrap confidence intervals on the independent test set.

**Results:**

In multivariable logistic regression, no individual continuous morphometric predictor reached statistical significance for any endpoint. After correction of the model-selection procedure, predictive performance proved endpoint-specific rather than uniformly strong. For composite chondromalacia, discrimination remained weak. For the ACL injury/patellar bone bruise composite, machine-learning models showed only modest improvement in point estimates over logistic regression, but confidence intervals for differences crossed zero. The strongest reproducible signal was observed for patellar retinacular lesions. After correction of model selection and bootstrap-based uncertainty estimation, a morphometric-only CatBoost model achieved an AUC of 0.85 (95% CI 0.68–0.97) and balanced accuracy of 0.79 (95% CI 0.61–0.94), while a morphometric-only LightGBM model achieved an AUC of 0.84 (95% CI 0.64–0.97) and balanced accuracy of 0.76 (95% CI 0.59–0.88).

**Discussion:**

In adolescents, routine patellofemoral morphometrics do not provide uniformly strong diagnostic discrimination across all MRI-defined knee pathologies studied here. Their most convincing predictive value was observed for patellar retinacular lesions, whereas performance for composite chondromalacia and the ACL injury/patellar bone bruise composite remained limited. These findings support a narrower interpretation of clinical utility and justify further validation in larger external cohorts.

## 1 Introduction

The patellofemoral joint (PFJ) is a complex anatomical and biomechanical structure that plays a critical role in knee motion, load transmission, and stability. In adolescents, developmental variability in trochlear shape, patellar height, and alignment may predispose individuals to structural abnormalities such as chondromalacia, patellar instability, and retinacular injury, which are among the most frequent causes of anterior knee pain in this age group. Early identification of anatomically unfavorable morphometric profiles may improve diagnostic stratification and help refine clinical decision-making in this population (Pfirrmann et al., 2000; Dejour et al., 1994).

Magnetic resonance imaging (MRI) enables comprehensive morphometric evaluation of the PFJ by providing accurate and reproducible assessment of anatomical indices such as Wiberg patellar type, trochlear depth, tibial tubercle-trochlear groove (TT-TG) distance, and patellar height ratios, including the Insall-Salvati and Caton-Deschamps indices. These parameters have been associated with maltracking, instability, and focal cartilage wear (Schoettle et al., 2006; Goldberg, 1991; Wibeeg, 1941; Insall and Salvati, 1971). However, in routine clinical practice, no single morphometric marker has shown sufficient standalone diagnostic accuracy. This limitation is particularly relevant in adolescents, in whom skeletal immaturity introduces both anatomical variability and additional uncertainty in the interpretation of imaging findings (Swain et al., 2018).

Several studies have explored the diagnostic potential of MRI-based morphometric, radiomic, or image-derived data for knee pathology prediction using machine learning or related statistical approaches. Nagawa et al. combined three-dimensional MRI statistical shape analysis with machine learning and reported strong discrimination for patellofemoral instability in young patients, while Gudas et al. related patellar morphology to arthroscopy-confirmed degeneration (Nagawa et al., 2024; Gudas et al., 2018). Other groups have used radiomics or deep learning to classify knee osteoarthritis and multiple knee abnormalities, and to automate PFJ morphometry or detection of trochlear dysplasia (Qiu et al., 2024; Barbosa et al., 2024; Lyu et al., 2025; Liu et al., 2023; Dunnhofer et al., 2021). More recently, modern tabular-learning approaches, including gradient boosting and tabular foundation models, have shown strong performance on structured clinical prediction tasks, further emphasizing the rapid evolution of machine-learning methodology for tabular biomedical data (Kwon et al., 2025; Hollmann et al., 2025; Qu et al., 2025). Nevertheless, few approaches have focused specifically on adolescents, and even fewer have evaluated whether routinely measured PFJ morphometric indices alone can provide meaningful diagnostic signal in this age group.

The rationale for studying routine morphometric indices separately from radiomics or full-image deep learning is both practical and scientific. PFJ morphometric parameters are interpretable, already embedded in standard musculoskeletal MRI workflows, and directly linked to established anatomical concepts such as trochlear dysplasia, patellar height abnormality, and maltracking. In contrast, radiomics and deep-learning pipelines generally require additional feature extraction, computational infrastructure, or image-level model development that may be more difficult to integrate into routine clinical workflows. Thus, even if morphometrics do not capture the full representational richness of image-wide models, they may offer a more transparent and operationally feasible route to diagnostic support in adolescent knee imaging.

In this context, the present study was designed as a pilot diagnostic modeling analysis of whether routine MRI-based PFJ morphometric parameters can predict three MRI-defined structural pathology endpoints in adolescents: composite chondromalacia, a composite endpoint of ACL injury or patellar bone bruise, and patellar retinacular lesion. We compared a conventional multivariable logistic regression baseline with machine-learning approaches and modern tree-based tabular comparators. Rather than assuming uniformly strong predictive value, our aim was to determine for which specific endpoints routine PFJ morphometrics retain clinically meaningful diagnostic information and for which they do not.

## 2 Materials and methods

### 2.1 Study design and population

This retrospective single-center study was conducted at a university-affiliated radiology department and included 168 adolescent patients (97 females [57.7%], 71 males [42.3%]) who underwent knee MRI between January 2018 and December 2024. The study was approved by the institutional ethics committee (approval no. 01716/15) and conducted in accordance with the Declaration of Helsinki; given the retrospective design and use of anonymized data, the requirement for written informed consent was waived.

Patients were referred for MRI because of anterior knee pain, suspected patellar instability, or structural abnormalities. Inclusion criteria were age 12–18 years and clinical symptoms suggestive of anterior knee pain, instability, or structural pathology. Exclusion criteria comprised prior knee surgery, inflammatory arthritis, and systemic musculoskeletal disorders. All eligible examinations during the study period were included; no *a priori* power calculation was performed, and the sample size was determined by case availability.

### 2.2 MRI acquisition

All examinations were performed on a 1.5-T MRI scanner (GE Medical Systems) with a dedicated knee coil. The protocol included axial, coronal, and sagittal T1-weighted, T2-weighted, and proton-density fat-saturated sequences with 3-mm slice thickness and 0.5-mm interslice gap.

### 2.3 Morphometric measurements

Thirteen patellofemoral morphometric parameters were assessed on MRI, either measured directly or derived from linear dimensions according to established formulas (Pfirrmann et al., 2000; Dejour et al., 1994; Schoettle et al., 2006; Goldberg, 1991; Wibeeg, 1941; Insall and Salvati, 1971; Swain et al., 2018). Measurements were obtained on proton-density fat-saturated axial and sagittal sequences with the knee flexed at approximately 30°. Parameters included medial and lateral trochlear facet lengths and heights, sulcus depth, tibial tubercle-trochlear groove (TT-TG) distance, patellar length, patellar articular surface length, patellar tendon length, trochlear facet asymmetry, Pfirrmann trochlear depth, and patellar height indices (Insall-Salvati index [ISI] and Caton-Deschamps index [CDI]) (Pfirrmann et al., 2000; Dejour et al., 1994; Schoettle et al., 2006; Goldberg, 1991; Wibeeg, 1941; Insall and Salvati, 1971; Swain et al., 2018).

All morphometric indices were independently measured by two experienced musculoskeletal radiologists, each with more than 10 years of experience in pediatric knee MRI, in separate sessions, blinded to clinical information and to each other’s results. Inter-rater reliability was evaluated using a two-way mixed-effects, absolute-agreement intraclass correlation coefficient (ICC) for continuous variables and Cohen’s *κ* for categorical variables. ICC values for continuous measures ranged from 0.19 to 0.99, while Cohen’s κ for categorical assessments was 0.82, indicating substantial agreement.

Categorical assessments comprised Wiberg patellar type (I-III) and Dejour trochlear dysplasia classification (types A-D vs. X) (Nagawa et al., 2024; Dejour et al., 1994). For the multivariable logistic regression baseline, five continuous morphometric predictors were entered simultaneously: trochlear facet asymmetry, Pfirrmann trochlear depth, tibial tubercle-trochlear groove distance, Insall-Salvati index, and Caton-Deschamps index. Categorical morphology variables were analyzed descriptively but were not retained in the final logistic baseline because sparse categories yielded unstable estimates in this sample.

### 2.4 Outcome definitions

For modeling, three binary MRI endpoints were constructed:

Composite chondromalacia: any chondral lesion involving the medial facet, lateral facet, or patellar ridge (0 = absent, 1 = present).
ACL/patellar bone bruise composite: presence of either ACL injury or patellar bone bruise (0 = neither, 1 = ACL injury or patellar bone bruise).
Patellar retinacular lesion: presence of a medial or lateral retinacular lesion (0 = absent, 1 = present).

These endpoints were used identically in multivariable logistic regression and machine-learning analyses.

### 2.5 Machine-learning workflow

Supervised machine-learning analyses were performed in two stages. First, an exploratory candidate-pipeline framework was implemented using the open-source FeAture Explorer (FAE) Python package, which incorporates NumPy, pandas, and scikit-learn (Song, 2020; Song et al., 2020). Within this framework, candidate models were generated by combining different normalization, preprocessing, feature-selection, and classification steps. Normalization options included MinMax scaling (0–1), Z-score standardization, and mean normalization (−0.5 to 0.5). Preprocessing strategies comprised principal component analysis (PCA) or removal of highly correlated features (Pearson correlation coefficient > 0.97). Feature selection used one of four methods: ANOVA, Kruskal-Wallis, recursive feature elimination (RFE), or Relief. The selected features (2–8 per model) were then input into classifiers including support vector machine (SVM), linear discriminant analysis (LDA), logistic regression (LR), AdaBoost, Gaussian process (GP), multilayer perceptron (MLP), random forest (RF), least absolute shrinkage and selection operator (LASSO), decision tree (DT), or naïve Bayes (NB). In total, 1,680 unique pipelines were specified for each endpoint.

Second, leakage-free comparator analyses were additionally performed using modern tree-based tabular models, specifically CatBoost, LightGBM, and XGBoost. TabPFN was also evaluated as an exploratory foundation-model comparator because it was specifically developed for small-to-medium tabular datasets, which better matched the present cohort size (Hollmann et al., 2025).

Patients were chronologically split into training (*n* = 83), validation (*n* = 35), and test (*n* = 50) sets, preserving the temporal order of examinations. Candidate models were developed using the training set, while model ranking and threshold selection were based exclusively on validation-set performance. Final performance was then evaluated once on the independent test set. To avoid information leakage, no test-set information was used for model ranking, threshold tuning, or comparator selection. When probabilistic models required dichotomization, the operating threshold was derived from validation-set performance using the Youden index and then applied unchanged to the test set.

Performance metrics included area under the receiver-operating-characteristic curve (AUC), accuracy, balanced accuracy, sensitivity, specificity, F1 score, Youden index, and Matthews correlation coefficient (MCC).

**Figure 1:**
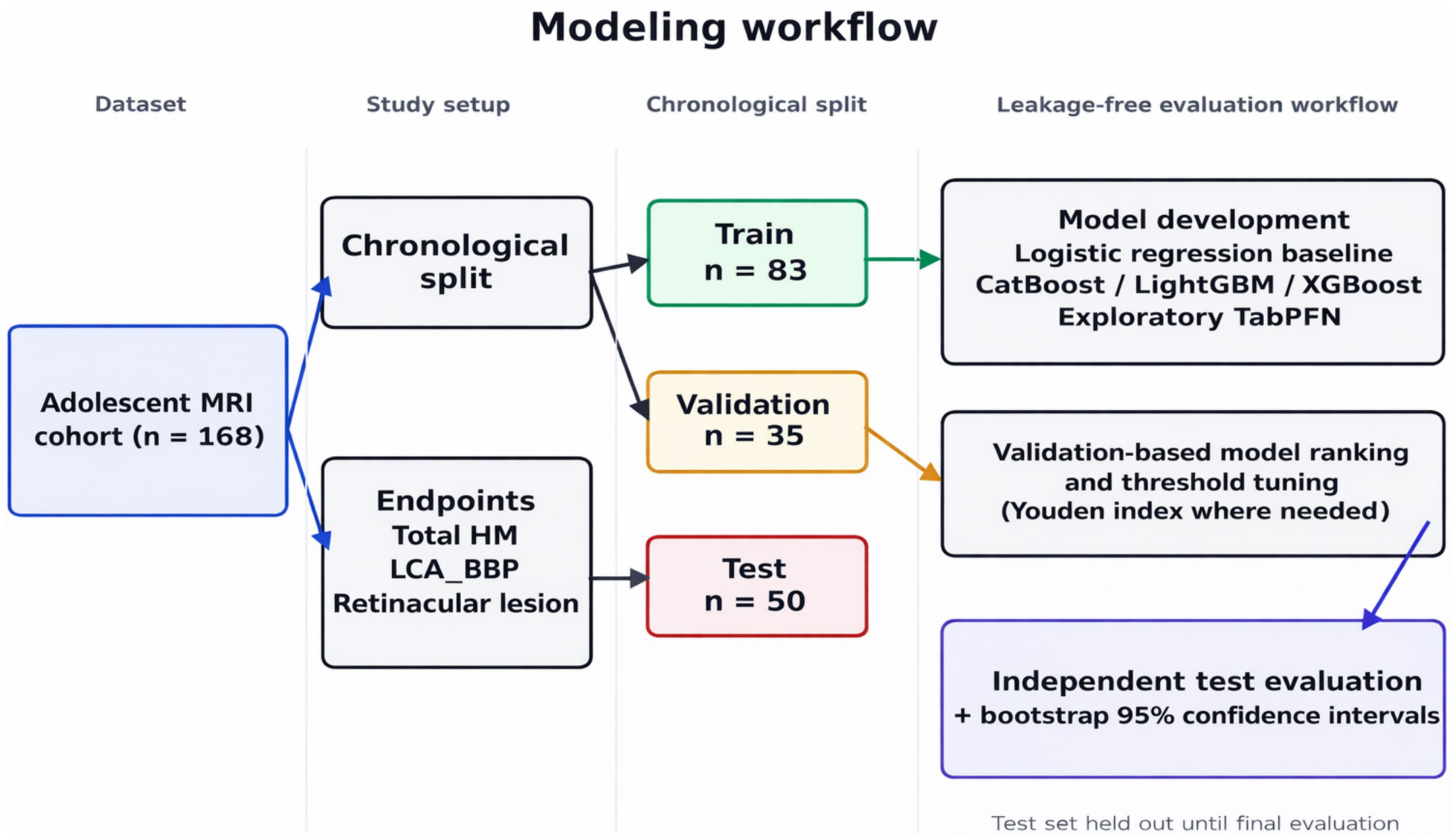
MRI cohort modeling workflow.

The overall modeling workflow, including the chronological split, leakage-free validation strategy, and independent test evaluation, is summarized in Figure 1.

### 2.6 Supplementary feature-stability assessment

As a supplementary interpretability analysis, feature stability was summarized across the strongest morphometric-only models for the retinacular-lesion endpoint, which showed the most robust predictive signal in the study. The summary focused on model consistency across the strongest corrected comparator models rather than on pipeline-wide inclusion frequencies from the superseded test-selected workflow.

### 2.7 Statistical analysis

Multivariable binary logistic regression models were fitted for the three endpoints using IBM SPSS Statistics (version 26.0; IBM Corp., Armonk, NY). For each model, odds ratios (ORs) with 95% confidence intervals (CIs), Wald statistics, and *p* values were reported. Model-level sensitivity, specificity, and accuracy were derived from classification tables; for the ACL/patellar bone bruise composite, the operating point was additionally optimized using the Youden index on the ROC curve. Calibration was assessed with the Hosmer-Lemeshow test, and overall fit was summarized with Cox & Snell and Nagelkerke R^2^. No separate correlation tests were performed prior to regression analysis. A two-sided *p* < 0.05 was considered statistically significant.

For the machine-learning analyses (scripts written in Python 3.14.0 and executed in Visual Studio Code version 1.116), 95% confidence intervals for discrimination and classification metrics were estimated on the independent test set by nonparametric bootstrap resampling with replacement. Bootstrap distributions were also used to estimate confidence intervals for differences in performance between the logistic regression baseline and the strongest machine-learning models (Efron and Tibshirani, 1994).

## 3 Results

### 3.1 Study population and diagnoses

Table 1 summarizes the demographic and clinical characteristics of the 168 adolescent patients. Mean age was 15.5 ± 1.7 years, with a female predominance (57.7%) and a balanced distribution of left and right knees (51.2% vs. 48.8%). Composite chondromalacia (Total HM) was the most frequent MRI-defined endpoint, present in 29.8% of patients, followed by patellar retinacular lesions in 14.3%, the ACL/patellar bone bruise composite in 12.5%, anterior cruciate ligament (ACL) injuries in 8.9%, and patellar bone bruises in 3.6%. Diagnoses were not mutually exclusive and frequently overlapped.

**Table 1:** General characteristics of the study Cohort (*n* = 168).

Variable	Value
General parameters	
Sex, male/female	71/97 (42.3%/57.7%)
Mean age ± SD, years	15.5 ± 1.7
Age range, years	13–18
Mean height ± SD, cm	165.0 ± 9.0
Mean weight ± SD, kg	58.8 ± 9.4
Mean BMI ± SD	21.5 ± 1.9
Injured knee side, left/right	86/82 (51.2%/48.8%)
Number of previous injuries of the same knee, mean ± SD	0.39 ± 0.62
MRI-defined endpoints/diagnoses	
Composite chondromalacia (Total HM)	50 patients (29.8%)
Patellar retinacular lesion	24 patients (14.3%)
ACL injury	15 patients (8.9%)
Patellar bone bruise	6 patients (3.6%)
ACL injury + patellar bone bruise composite (LCA_BBP)	21 patients (12.5%)
Patellar and trochlear morphology	
Wiberg type I/II/III/IV	17 (10.1%)/126 (75.0%)/24 (14.3%)/1 (0.6%)
Dejour X (normal)	87 (51.8%)
Dejour A–D (dysplastic)	81 (48.2%)

This table summarizes the demographic, anatomical, and clinical features of the adolescent patients included in the study. Mean values are reported with standard deviations.

In terms of anatomical morphology, Wiberg type II was most prevalent (75.0%), followed by type III (14.3%), type I (10.1%), and type IV (0.6%). According to Dejour classification, trochlear dysplasia (types A-D) was present in 48.2% of patients, while 51.8% were classified as normal (type X).

### 3.2 Morphometric measurements and reliability

Interrater analysis across all 13 morphometric parameters demonstrated overall high reproducibility between the two radiologists, with intraclass correlation coefficient (ICC) values for continuous measurements ranging from 0.19 to 0.99. Measurements of trochlear and patellar morphology showed particularly consistent results, with ICC values above 0.88 for medial and lateral trochlear facet lengths, trochlear facet asymmetry, sulcus depth, lateral trochlear facet height, tibial tubercle-trochlear groove (TT-TG) distance, and patellar height indices (Insall-Salvati and Caton-Deschamps). Moderate reliability was observed for medial trochlear facet height (ICC = 0.80) and patellar length (ICC = 0.65), whereas Pfirrmann trochlear depth exhibited the lowest reproducibility (ICC = 0.19), reflecting its sensitivity to slice selection and identification of the trochlear sulcus. Categorical assessments of patellar and trochlear morphology also showed strong concordance, with Cohen’s *κ* = 0.82 for both Wiberg type and Dejour classification.

### 3.3 Multivariable logistic regression

Table 2 summarizes the multivariable logistic regression coefficients and model-level performance for the three binary MRI endpoints: composite chondromalacia, the composite endpoint of ACL injury or patellar bone bruise, and patellar retinacular lesion. Across all three models, none of the evaluated continuous morphometric predictors reached statistical significance (all *p* > 0.05). Pseudo-R^2^ values were low, indicating limited explanatory power of the baseline models, with Cox and Snell/Nagelkerke R^2^ values of 0.031/0.045 for composite chondromalacia, 0.038/0.072 for the ACL/patellar bone bruise composite, and 0.073/0.131 for patellar retinacular lesions.

**Table 2:** Multivariable logistic regression coefficients and model-level performance for the three MRI-defined endpoints.

Outcome	Predictor	Wald	*p* value	OR [Exp(B)]	95% CI
Composite chondromalacia	Trochlear facet asymmetry	0.817	0.366	0.339	0.032–3.544
	Pfirrmann trochlear depth	1.834	0.176	0.894	0.760–1.052
	TT–TG distance	0.624	0.430	0.970	0.899–1.046
	Insall–Salvati index	0.407	0.523	1.872	0.273–12.830
	Caton–Deschamps index	0.225	0.635	0.585	0.064–5.363
ACL injury/patellar bone bruise composite	Trochlear facet asymmetry	1.706	0.191	0.095	0.003–3.251
	Pfirrmann trochlear depth	2.063	0.151	1.165	0.946–1.435
	TT–TG distance	0.632	0.427	1.042	0.942–1.152
	Insall–Salvati index	1.192	0.275	0.200	0.011–3.591
	Caton–Deschamps index	0.016	0.898	0.828	0.046–14.982
Patellar retinacular lesion	Trochlear facet asymmetry	0.471	0.493	0.339	0.015–7.450
	Pfirrmann trochlear depth	1.737	0.188	0.855	0.677–1.080
	TT–TG distance	1.551	0.213	1.062	0.966–1.168
	Insall–Salvati index	0.533	0.465	2.437	0.223–26.628
	Caton–Deschamps index	2.513	0.113	9.081	0.594–138.877

Model-level performance note: composite chondromalacia: AUC 0.61, sensitivity 4.0%, specificity 99.2%, accuracy 70.8%. ACL injury/patellar bone bruise composite: AUC 0.65, sensitivity 33.3%, specificity 92.5%, accuracy 85.1% at the Youden-optimized operating point. Patellar retinacular lesion: AUC 0.68, sensitivity 8.3%, specificity 100.0%, accuracy 86.9%. OR, odds ratio; CI, confidence interval; TT–TG, tibial tubercle–trochlear groove.

Despite the absence of statistically significant individual predictors, the models showed moderate discrimination but generally poor sensitivity. For composite chondromalacia, the logistic regression baseline achieved an AUC of 0.61, accuracy of 70.8%, sensitivity of 4.0%, and specificity of 99.2%. For the ACL/patellar bone bruise composite, the logistic regression baseline achieved an AUC of 0.65, accuracy of 85.1%, sensitivity of 33.3%, and specificity of 92.5% at the Youden-optimized operating point. For patellar retinacular lesions, the logistic regression baseline achieved an AUC of 0.68, accuracy of 86.9%, sensitivity of 8.3%, and specificity of 100.0%. Overall, these findings indicate that conventional logistic regression provided a limited baseline, driven largely by correct classification of negative cases rather than balanced discrimination across outcome classes (see Table 3).

**Table 3:** Machine-learning comparator performance on the independent test set.

Endpoint	Model	Feature block	AUC (95% CI)	Balanced accuracy (95% CI)	Sensitivity	Specificity	Accuracy	Interpretation
Composite chondromalacia (Total HM)	LightGBM	Clinical + morphometric	0.57 (0.41–0.73)	0.51 (0.46–0.58)	0.06	0.97	0.64	Weak discrimination; no robust improvement over logistic baseline
ACL injury/patellar bone bruise composite	CatBoost	Clinical + morphometric	0.67 (0.35–0.94)	0.64 (0.45–0.85)	0.38	0.91	0.82	Modest point-estimate improvement; confidence intervals overlap baseline
ACL injury/patellar bone bruise composite	CatBoost	Morphometric only	0.64 (0.36–0.92)	0.69 (0.50–0.88)	0.63	0.76	0.74	Similar alternative corrected model
Patellar retinacular lesion	CatBoost	Morphometric only	0.85 (0.68–0.97)	0.79 (0.61–0.94)	75.0%	83.3%	82.0%	Strongest reproducible corrected model after validation-only tie-break selection
Patellar retinacular lesion	LightGBM	Morphometric only	0.84 (0.64–0.97)	0.76 (0.59–0.88)	87.5%	64.3%	68.0%	Strong secondary corrected model with higher sensitivity but lower specificity and accuracy

Corrected machine-learning analyses used a leakage-free chronological split (training *n* = 83, validation *n* = 35, test *n* = 50), with model ranking and threshold selection based exclusively on validation-set performance. Confidence intervals were estimated by bootstrap resampling of the independent test set.

### 3.4 Machine-learning models

Machine-learning performance was evaluated using a chronological split with model ranking based exclusively on validation-set performance and final assessment on the independent test set. Under this framework, predictive performance differed substantially across endpoints. For composite chondromalacia, discrimination remained weak. The best corrected model, LightGBM using combined clinical and morphometric predictors, achieved an AUC of 0.57 (95% CI 0.41–0.73) and balanced accuracy of 0.51 (95% CI 0.46–0.58), which did not represent a robust improvement over the logistic regression baseline.

For the ACL injury/patellar bone bruise composite, machine-learning models showed modest improvement in point estimates compared with logistic regression, but bootstrap confidence intervals for differences versus logistic regression crossed zero. The best model by test-set AUC was CatBoost using combined clinical and morphometric predictors, with an AUC of 0.67 (95% CI 0.35–0.94), accuracy of 82%, sensitivity of 37.5%, specificity of 90.5%, and balanced accuracy of 0.64 (95% CI 0.45–0.85). A morphometric-only CatBoost model yielded a similar AUC of 0.64 (95% CI 0.36–0.92) and a slightly higher balanced accuracy of 0.69 (95% CI 0.50–0.88). Exploratory TabPFN benchmarking showed only modest discrimination for this endpoint (AUC 0.57, balanced accuracy 0.60 after validation-based threshold selection).

The strongest corrected signal was observed for patellar retinacular lesions. After validation-only tie-break selection among equally ranked CatBoost candidates, the final morphometric-only CatBoost model achieved an AUC of 0.85 (95% CI 0.68–0.97), balanced accuracy of 0.79 (95% CI 0.61–0.94), sensitivity of 75.0%, specificity of 83.3%, and accuracy of 82.0% on the independent test set. A morphometric-only LightGBM model also performed strongly, with an AUC of 0.84 (95% CI 0.64–0.97), balanced accuracy of 0.76 (95% CI 0.59–0.88), sensitivity of 87.5%, specificity of 64.3%, and accuracy of 68.0%. Exploratory TabPFN evaluation yielded moderate discrimination for retinacular lesions (AUC 0.74, balanced accuracy 0.67, sensitivity 75.0%, specificity 59.5%), but it did not outperform the best corrected gradient-boosting models. In paired bootstrap comparisons against the logistic regression baseline, confidence intervals for AUC differences crossed zero, whereas confidence intervals for balanced-accuracy differences remained above zero.

Exploratory additional comparator results for TabPFN across all three endpoints are summarized in Supplementary Table S2.

**Figure 2:**
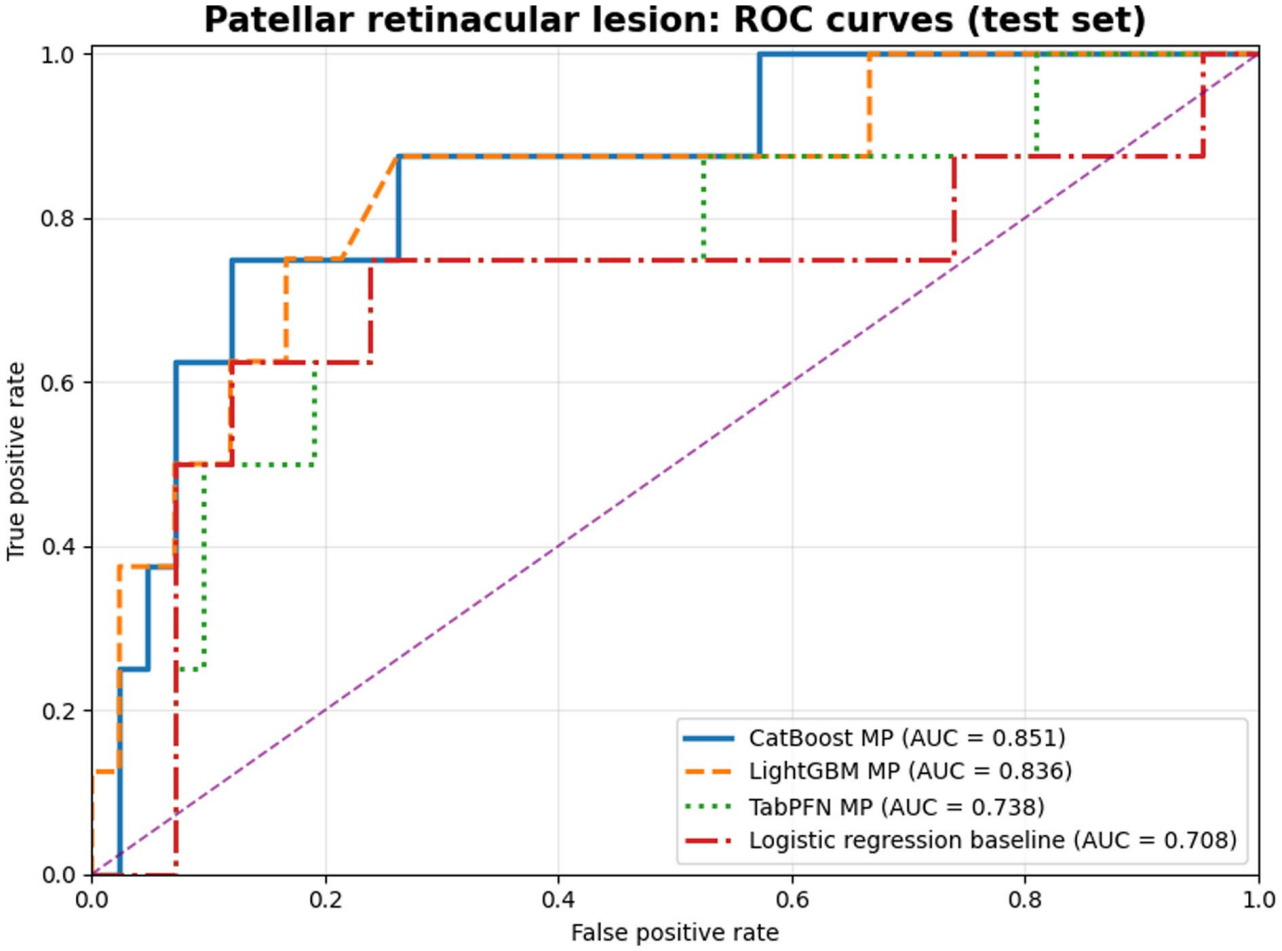
ROC curve comparison graph for patellar retinacular lesion.

Receiver-operating-characteristic curves comparing the logistic regression baseline with the final validation-selected retinacular-lesion comparator models are shown in Figure 2. A supplementary feature-stability summary for the retinacular-lesion endpoint is provided in Supplementary Table S1 and shows that all 13 morphometric predictors were retained across the strongest corrected morphometric-only comparator models.

## 4 Discussion

This study evaluated whether routine MRI-based patellofemoral joint morphometric parameters can support diagnostic modeling of selected adolescent knee pathologies and whether machine-learning methods offer meaningful advantages over a conventional multivariable logistic regression baseline under leakage-free evaluation. The results show a more constrained picture than initially thought. Predictive performance was not uniformly strong across the three modeled endpoints. Instead, the diagnostic value of routine PFJ morphometrics proved endpoint-specific: weak for composite chondromalacia, modest for the ACL injury/patellar bone bruise composite, and most convincing for patellar retinacular lesions.

This interpretation is important both methodologically and clinically. By restricting model ranking and threshold selection to validation-set performance, the apparent advantage of machine learning across all outcomes was attenuated. Only retinacular-lesion prediction retained a stronger signal relative to the logistic regression baseline, particularly with validation-selected morphometric-only CatBoost and LightGBM models. However, bootstrap confidence intervals for differences in AUC versus logistic regression crossed zero, whereas confidence intervals for differences in balanced accuracy remained above zero. Accordingly, the present study supports a narrower conclusion that routine PFJ morphometrics may be most informative for selected structural outcomes and that the main reproducible advantage of the corrected tree-based models lies in threshold-dependent classification performance rather than unequivocally superior discrimination.

The present findings also help clarify the role of routine morphometric indices in relation to more complex image-based approaches. Prior studies using statistical shape analysis, radiomics, or deep learning have reported promising performance for patellofemoral instability, osteoarthritis, and automated PFJ characterization. Nagawa et al. combined three-dimensional MRI statistical shape analysis with machine learning and reported strong discrimination for patellofemoral instability, while Barbosa et al. used a convolutional neural network to extract morphometric features and obtained moderate performance for trochlear dysplasia. Dunnhofer et al. improved classification of knee disorders using pyramidal convolutional neural networks, and radiomics-based studies have also shown encouraging results for osteoarthritis and multi-label abnormality detection (Nagawa et al., 2024; Barbosa et al., 2024; Dunnhofer et al., 2021; Qiu et al., 2024; Cui et al., 2023; Tuya et al., 2023). However, those approaches rely on richer image representations than the manually or semi-manually derived indices used here. Our results suggest that routine PFJ morphometrics alone do not provide uniformly strong discrimination across all endpoints, but can still retain clinically relevant predictive value for retinacular lesions, particularly in tree-based models that improved balanced classification performance under leakage-free evaluation. This remains important because morphometric indices are interpretable, already familiar to musculoskeletal radiologists and orthopedic clinicians, and comparatively easy to integrate into structured MRI reporting.

The analysis also highlights the limitations of conventional linear modeling in anatomically heterogeneous adolescent populations. Although logistic regression remains a reasonable and interpretable baseline, it assumes relatively simple relationships between predictors and outcomes and may underperform when multiple weak anatomical signals interact nonlinearly. In our analyses, this limitation was most evident for patellar retinacular lesions, where validation-selected tree-based models showed higher balanced accuracy than the logistic baseline, whereas for the other two endpoints the gains were either small or non-robust. At the same time, because paired bootstrap confidence intervals for AUC differences crossed zero, the evidence for superior discrimination should be interpreted cautiously. This pattern suggests that the relationship between PFJ morphology and structural pathology is not uniform across outcomes and may depend on endpoint-specific interaction structure rather than on any single dominant morphometric variable.

The relatively weak performance for composite chondromalacia is also consistent with the likelihood that cartilage abnormalities in adolescents are influenced by factors beyond static PFJ morphology alone, including activity level, biomechanics, and tissue-level processes not captured by the present morphometric measures (Gudas et al., 2018; Arendt and Dejour, 2013; Nelitz et al., 2017).

Interobserver reliability for patellofemoral morphometric parameters was generally high, confirming that most measurements can be obtained consistently by experienced musculoskeletal radiologists. ICC values were excellent for key parameters such as trochlear facet lengths, TT-TG distance, and patellar height indices, while lower reproducibility was observed for Pfirrmann trochlear depth and patellar length, parameters known to be sensitive to slice selection and incomplete ossification (Pfirrmann et al., 2000; Nelitz et al., 2017). Importantly, this lower reproducibility is unlikely to have materially influenced the modeling results, given that the machine-learning approaches operated on a multivariate feature set. Categorical assessments of Wiberg patellar type and Dejour classification also demonstrated substantial agreement, with *κ* values consistent with previous MRI-based studies (Lippacher et al., 2012). The strongest models for retinacular lesions were consistent in operating on the same morphometric predictor block, suggesting that the retained signal was not driven by an unstable single-feature selection artifact but instead by the joint structure of routine PFJ measurements considered together.

From a translational perspective, the present findings support cautious rather than expansive clinical interpretation. These results do not justify broad claims of automated prognostication or generalized risk stratification across all modeled adolescent knee pathologies. Rather, they suggest that routine PFJ morphometrics may assist structured diagnostic support for selected MRI-defined outcomes, particularly retinacular lesions, while remaining insufficient as standalone predictors for others. As automated landmark detection and morphometric extraction continue to improve, such indices may eventually be incorporated into radiology decision-support tools, but this should be pursued as an incremental diagnostic aid rather than as a replacement for comprehensive image interpretation or clinical judgment. In practice, such models may be most useful as an adjunct tool for structured reporting, helping to highlight morphometric patterns associated with specific structural findings rather than directly altering first-line clinical management. In this context, it is also notable that modern tabular-learning methods are evolving rapidly. Tree-based models remained the strongest corrected comparators in the present study, while exploratory TabPFN benchmarking showed moderate but not superior performance; TabICL was not prioritized as a primary benchmark because it was developed mainly for substantially larger tabular datasets than the present 168-patient cohort (Hollmann et al., 2025; Qu et al., 2025; Kwon et al., 2025).

This study has several limitations. First, it was a retrospective single-center analysis with a modest sample size and class imbalance for some endpoints, which limits precision and may increase instability of performance estimates. Second, no external validation cohort was available, and MRI rather than arthroscopy or longitudinal clinical follow-up served as the reference standard. Third, morphometric measurements were manual, and although most indices showed high reproducibility, some parameters, particularly Pfirrmann trochlear depth, showed limited inter-rater reliability. Finally, even the best corrected models demonstrated endpoint-specific rather than universal utility, indicating that PFJ morphometry alone is unlikely to capture the full biological complexity of adolescent knee pathology. Model calibration was not formally assessed in the present analysis, as the primary objective was to evaluate discriminative performance across candidate models in a relatively small cohort; future studies with larger datasets should incorporate calibration analysis to further support clinical applicability. Accordingly, the present analysis should be interpreted as a pilot study designed to explore whether routinely available morphometric parameters contain sufficient diagnostic signal to justify further investigation in larger cohorts.

Overall, this study is a diagnostic modeling analysis showing that routine PFJ morphometrics contain outcome-specific rather than universal predictive signal in adolescents, with the most robust evidence observed for patellar retinacular lesions. As a pilot diagnostic modeling study, these findings require confirmation in larger, ideally multicenter cohorts before clinical translation can be considered. Future work should prioritize external multicenter validation, more systematic integration with automated morphometric extraction, and comparison with richer image-based approaches to clarify when routine morphometric modeling is sufficient and when more complex representations are necessary.

## Data Availability

The datasets presented in this article are not readily available because the raw anonymized data are not publicly available due to patient confidentiality and institutional restrictions. Access may be granted to qualified researchers upon reasonable request to the corresponding author, subject to institutional approvals and a data-use agreement. Requests to access the datasets should be directed to dusan.spasic@beograd.com.
