# Supplementary material for: MRI-based morphometric analysis of the patellofemoral joint: diagnostic modeling of knee pathologies in adolescents
Source: Front Artif Intell. 2026 May 22;9:1808422. doi: 10.3389/frai.2026.1808422 (PMC13236875; doi:10.3389/frai.2026.1808422)
Supplement: Supplementary file 1 [file Table_1.DOCX]

Table S1. Feature-stability summary across the strongest corrected leakage-free morphometric-only models for patellar retinacular lesion prediction

| **Morphometric predictor** | **CatBoost (MP)** | **LightGBM (MP)** | **XGBoost (MP)** | **TabPFN (MP)** | **Stability interpretation** |
| --- | --- | --- | --- | --- | --- |
| Medial patellar facet length | Included | Included | Included | Included | Retained across all corrected morphometric-only comparators |
| Lateral patellar facet length | Included | Included | Included | Included | Retained across all corrected morphometric-only comparators |
| Medial trochlear facet height | Included | Included | Included | Included | Retained across all corrected morphometric-only comparators |
| Lateral trochlear facet height | Included | Included | Included | Included | Retained across all corrected morphometric-only comparators |
| Sulcus depth | Included | Included | Included | Included | Retained across all corrected morphometric-only comparators |
| Tibial tubercle-trochlear groove distance | Included | Included | Included | Included | Retained across all corrected morphometric-only comparators |
| Medial trochlear depth component (A) | Included | Included | Included | Included | Retained across all corrected morphometric-only comparators |
| Lateral trochlear depth component (B) | Included | Included | Included | Included | Retained across all corrected morphometric-only comparators |
| Central trochlear depth component (C) | Included | Included | Included | Included | Retained across all corrected morphometric-only comparators |
| Trochlear facet asymmetry | Included | Included | Included | Included | Retained across all corrected morphometric-only comparators |
| Pfirrmann trochlear depth (TD, mm) | Included | Included | Included | Included | Retained across all corrected morphometric-only comparators; interpret cautiously because inter-rater reproducibility was lower than for most other morphometric indices |
| Insall-Salvati index | Included | Included | Included | Included | Retained across all corrected morphometric-only comparators |
| Caton-Deschamps index | Included | Included | Included | Included | Retained across all corrected morphometric-only comparators |
| **Abbreviations:** MP, morphometric predictors only; TT-TG, tibial tubercle-trochlear groove.  **Table note:** This supplementary table summarizes feature stability across the strongest leakage-free morphometric-only comparator models for patellar retinacular lesion prediction, the endpoint that showed the most robust predictive signal in the analysis. Because the strongest corrected models were refit using the full morphometric predictor block rather than sparse wrapper-selected subsets, model consistency across corrected comparators is shown here instead of pipeline-wide inclusion percentages from the superseded test-selected workflow. TabPFN was evaluated as an exploratory foundation-model comparator using the same morphometric block and a validation-derived threshold. | | | | | |

Table S2. Exploratory additional comparator performance

| **Endpoint** | **Model** | **Feature block** | **AUC** | **Balanced accuracy** | **Sensitivity** | **Specificity** | **Accuracy** |
| --- | --- | --- | --- | --- | --- | --- | --- |
| Patellar retinacular lesion | TabPFN | Morphometric only | 0.738 | 0.673 | 0.750 | 0.595 | 0.620 |
| ACL injury / patellar bone bruise composite | TabPFN | Morphometric only | 0.574 | 0.595 | 0.500 | 0.690 | 0.660 |
| Composite chondromalacia (Total HM) | TabPFN | Morphometric only | 0.512 | 0.434 | 0.056 | 0.812 | 0.540 |
| **Table note:** TabPFN results are shown after validation-based threshold optimization using the Youden index. These exploratory results did not outperform the strongest corrected gradient-boosting models. | | | | | | | |
